# Plantar Stress-Related Injuries in Male Basketball Players: Variations on Plantar Loads during Different Maximum-Effort Maneuvers

**DOI:** 10.1155/2018/4523849

**Published:** 2018-04-24

**Authors:** Yiyang Chen, Jing Xian Li, Youlian Hong, Lin Wang

**Affiliations:** ^1^School of Kinesiology, Shanghai University of Sport, Shanghai, China; ^2^Key Laboratory of Exercise and Health Science of the Ministry of Education, Shanghai University of Sport, Shanghai, China; ^3^School of Human Kinetics, University of Ottawa, Ottawa, ON, Canada; ^4^Department of Sports Medicine, Chengdu Sports University, Chengdu, China

## Abstract

This study aims to compare the insole load of three maximum-effort cutting tasks in basketball. Sixteen male basketball players were recruited to participate in the study. The Pedar Mobile system was used to record the insole plantar load distribution during three cutting tasks (45° cutting, 90° cutting, and sideward cutting). The peak pressures (PP) and maximum force (MF) at the total foot and at each foot mask were used in data analysis. ANOVA with repeated measures was employed to investigate the differences in the measures among these cutting tasks. At the total foot, the highest MF value was showed when performing sideward cutting. At the heel, the highest PP and MF were found when performing 90° cutting. The PP and MF were lower when performing 90° cutting than when conducting 45° and sideward cuttings at the medial midfoot and the central forefoot. Furthermore, the MF value was lower when performing 45° cutting than when conducting sideward cutting at the medial midfoot and the central forefoot. These findings corroborate the fact that plantar loads differed during the three maximum-effort cutting maneuvers. Differences in the plantar loads for different cutting may be potential risks for overuse-related injuries to the lower extremities of basketball players.

## 1. Introduction

Basketball has been one of the most popular sports around the world, with more than 450 million players worldwide [1]. The sport is also one of the leading causes of injuries [2]. The overall rate of injury is 9.9 per 1000 athlete-exposures for games and 4.3 per 1000 athlete-exposures in male basketball players [3]. The overall rate of injury is 7.7 per 1000 athlete-exposures for games and 4.0 per 1000 athlete-exposures in female basketball players [3, 4]. More than 60% of all injuries are to the lower extremities in male and female basketball players [3, 4]. Special footwork characteristics contribute to high sport injuries in basketball [2]. Basketball is an intermittent activity, characterized by combining movements of high intensity interspersed with short periods of low intensity or rest [5]. Basketball footwork is composed of special maneuver patterns, including lateral shuffling, sprinting, jumping, jogging, and cutting movements [6–8].

During offensive and defensive tasks in basketball, basketball players generally perform cutting in different directions. In an entire competitive basketball game, players spend 31% of their playing time performing cutting maneuvers, of which 20% are high-intensity activity [8]. Typical cutting movements characterize a sudden deceleration of the body, followed by acceleration in a new direction [9]. Cutting movements generate high shear force at the lower extremity [7, 9]. Excessive shear forces may induce large joint torque at the ankle and knee during the movement and are a potential risk factor for noncontact anterior cruciate ligament injury and ankle sprain [7, 9–12]. Furthermore, excessive and repetitive plantar stress may result in a high incidence of plantar stress-related foot injuries [13, 14]. Therefore, quantifications of plantar load characteristics on typical movements in basketball may provide valuable insight into sport injuries to the lower extremity.

In previous studies, characteristics of plantar loads have been investigated for cutting movements [9, 15, 16]. Wong et al. investigated plantar load distribution when players perform four soccer-typical tasks on turf and asserted that 45° cutting and sideward cutting demonstrate higher plantar peak pressure (PP) and pressure-time integral compared with running [16]. Sideward cutting showed more double-PP at the heel compared with running [15]. The players executed these soccer-typical tasks on turf while wearing soccer boots [15, 16]. Evidently, the playing court and footwear influence plantar load distribution [17]. Therefore, plantar loads on sport-typical movements should be measured using representative court and footwear. Up to now, little is known regarding plantar loads of typical movements on the basketball court. Several studies investigated plantar distribution on basketball-typical movements. These movements included free throw, jump stop shot, three-point shot, sprint, and shuffle. These studies have several limitations. Plantar loads were measured at the fifth metatarsal region [13, 14]. The participants executed the tasks while they were barefoot [5]. In a recent study, Cong et al. measured the plantar shear force of 45° cutting and sideward cutting at four anatomical sites using triaxial transducers in a laboratory setting [9]. They observed that the first and second metatarsal heads sustained relatively high pressure as well as shear stress and were expected to be susceptible to plantar tissue injuries [9]. Identifying the impact of forces and plantar pressure distribution characteristics during cutting in different directions may help reveal the risk factors related to sport injuries. To date, no quantitative information on the comparison of the entire plantar load characteristics during maximum-effort cutting tasks in different directions has been made available.

For the achievement of substantial insight into pressure loads and stress-related injuries in basketball, the plantar distribution of the entire foot during cutting in different directions should be investigated further. This present study aims to compare the insole load responses of three maximum-effort cutting tasks in basketball, namely, 45° cutting, 90° cutting, and sideward cutting. This study hypothesizes that plantar loads are higher while performing sideward cutting than when conducting 45° cutting and 90° cutting.

## 2. Materials and Methods

### 2.1. Participants

Sixteen healthy male recreational basketball players (age = 21.4 ± 2.4 years, body mass = 65.3 ± 9.5 kg, and body height = 1.71 ± 0.05 m) with a shoe size from 41 to 43 (European standard) were recruited to participate in the study. All the participants reported that they had been free from any lower extremity injury within the previous 6 months before the date of the study. Moreover, all of the subjects had the right foot as their dominant foot. Written informed consent forms were obtained, and the study was approved by the Ethics Committee of Shanghai University of Sport.

### 2.2. Measurements

The Pedar Mobile system (Novel GmbH, Munich, Germany), which contains 99 sensors in a matrix design, was used to record the insole plantar load distribution during the cutting test. The system was placed in between the insole of the shoes and the plantar surface of the foot. A belt was used to secure the data logger on the waist of each participant for data storage. The plantar pressure of the right foot was collected for analysis, and the sample frequency was set at 100 Hz. A synchronized video camera (9800, JVC Inc., Japan) with a sampling frequency of 100 Hz was used to record the feet movement of the participants. At the start of the data collection, the Pedar-X insole system generated a flash that was captured by the video camera as a synchronization signal. Subsequently, the video image was used to identify each right foot cutting maneuver of the Pedar-X records that was used in the data analysis.

All cutting tasks were performed on a standard wooden-based indoor basketball court. Each participant was given 15 minutes for the warm-up before the start of the cutting test and was asked to jog and stretch the large muscle groups, such as the quadriceps and the hamstrings, to reduce the chance of getting injured. Moreover, the participants could perform some basketball-related warm-ups, such as dribbling, shooting, and lay-ups. After the warm-up and prior to the data collection, they were allowed to perform as many practice trials as necessary to achieve the tasks as instructed.

**Figure 1:**
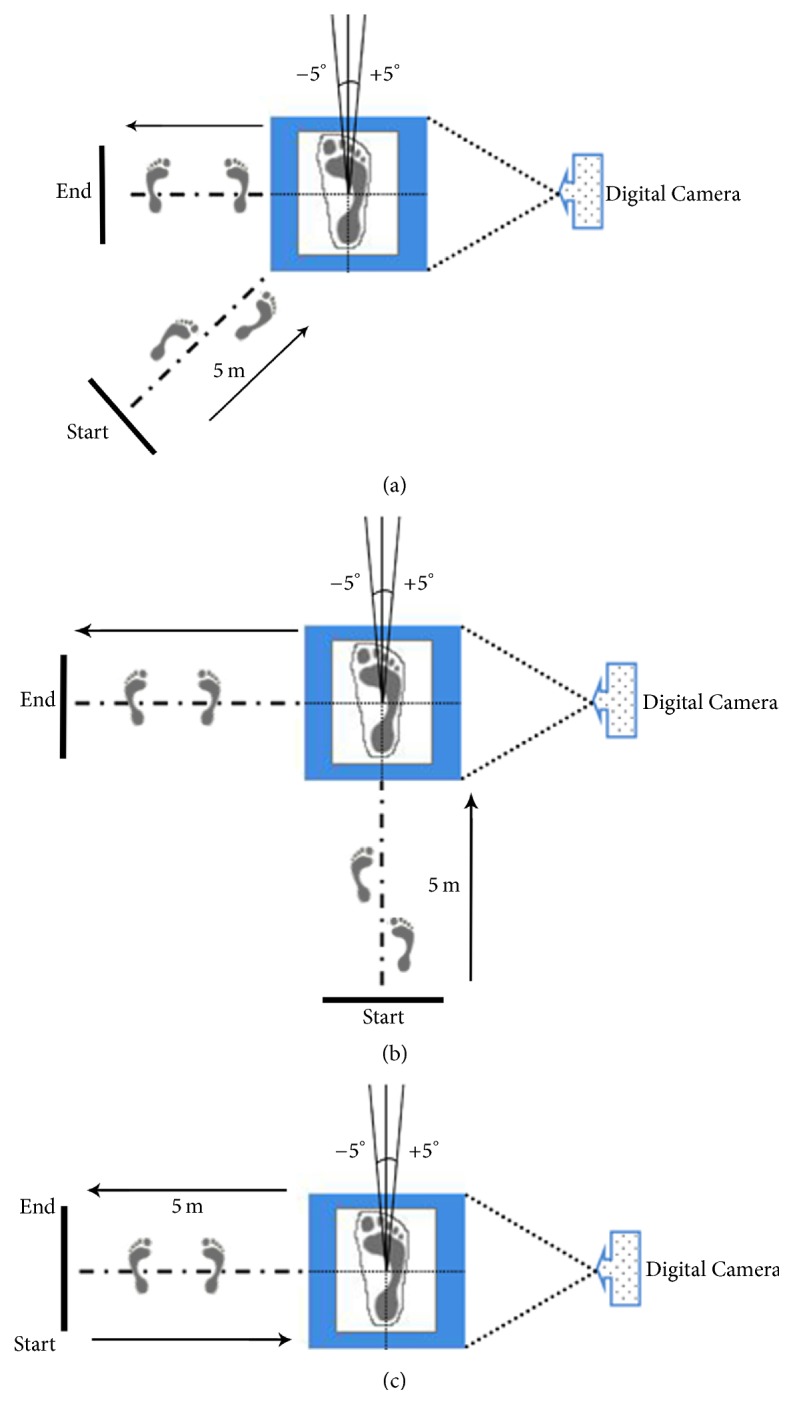
Experimental setup: (a) 45° cutting, (b) 90° cutting, and (c) sideward cutting.

Before the data collection, the participants put on a pair of thin black socks and wore the basketball shoes (Nike Air Barwin 318688 161). Subsequently, they were shown the cutting maneuver by a trained basketball coach and were asked to practice three types of motions as many times as necessary to perform the tasks as instructed. In the cutting test, the participants had 5 m run-up, and they were asked to perform the three types of maximum-effort cutting motions from a start position with their right foot landing on the target platform. For maximal 45° cutting, the participants started from the starting point and ran to the target platform, and, then, after performing the cutting at an approximately 45° angle, the participants performed sidesteps to the left end point. For maximal 90° cutting, they were instructed to sprint forward and performed maximum-effort cutting with their right foot hitting the platform onward and then cutting back to the end point. For maximal sideward cutting, they performed lateral side-cutting to the right and then took sidesteps to the end point. The plantar pressure data on the right foot were collected while the participants performed the cutting tasks at three different directions. Six successful trials in each direction were requested and presented in a randomized manner, and five were selected for the subsequent data analysis (Figure 1).

The insole was divided into nine areas for analyzing the plantar pressure. The nine areas were masked according to the features of the human foot, namely, the medial heel (M1), lateral heel (M2), medial midfoot (M3), lateral midfoot (M4), medial forefoot (M5), central forefoot (M6), lateral forefoot (M7), great toe (M8), and lesser toes (M9). The peak pressures (PP) and maximum force (MF) at the total foot and at each foot mask were extracted by employing the insole plantar pressure system.

### 2.3. Statistical Analyses

All the data were represented as mean ± standard deviation (SD). The homoscedasticity was verified using Levene's test. ANOVA with repeated measures (directions) was used to determine differences in each variable among the three cutting maneuvers. When ANOVA revealed significant direction interaction effects, such a technique with repeated measures was employed to investigate the differences in the measures among the directions. Significance was set to alpha < 0.05, and Bonferroni adjustment was used to correct multiple measurements.

## 3. Results 

Table 1 presents the mean and standard deviations of the PP and MF values.

**Figure 2:**
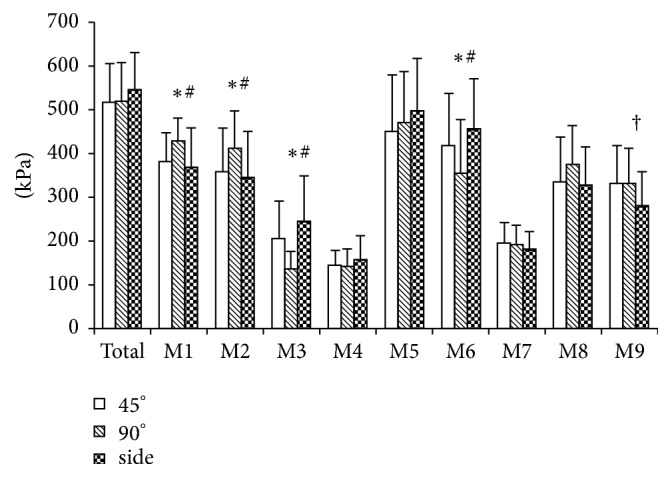
Comparison of peak pressure among three cutting maneuvers. (A) M1, medial heel; M2, lateral heel; M3, medial midfoot; M4, lateral midfoot; M5, medial forefoot; M6, central forefoot; M7, lateral forefoot; M8, great toe; M9, lesser toes. (B) 90° cutting versus 45° cutting, ^*∗*^*p* < 0.05; 90° cutting versus sideward cutting, ^#^*p* < 0.05; 45° cutting versus sideward cutting, ^†^*p* < 0.05.

The PP were higher when performing 90° cutting than when conducting 45° cutting and sideward cutting at the medial heel [*F*(2,14) = 4.955, *p* = 0.024; 90° versus 45°, *p* = 0.038, mean difference (MD) = 44.25 kPa, and 95% confidence interval for mean difference = 2.17–86.53 kPa; 90° versus side, *p* = 0.035, MD = 44.25 kPa, and 95% CI = 3.49–111.45 kPa] and the lateral heel [*F*(2,14) = 4.480, *p* = 0.031; 90° versus 45°, *p* = 0.041, MD = 54.07 kPa, and 95% CI = 1.86–106.29 kPa; 90° versus side, *p* = 0.043, MD = 68.10 kPa, and 95% CI = 1.86–134.34 kPa]. The PP were lower when performing 90° cutting than when conducting 45° cutting and sideward cutting at the medial midfoot [*F*(2,14) = 7.794, *p* = 0.005; 90° versus 45°, *p* = 0.022, MD = −66.98 kPa, and 95% CI = −125.32 to −8.65 kPa; 90° versus side, *p* = 0.004, MD = −106.72 kPa, and 95% CI = −178.99 to −34.46 kPa] and the central forefoot [*F*(2,14) = 10.90, *p* = 0.001; 90° versus 45°, *p* = 0.005, MD = −61.66 kPa, and 95% CI = −105.63 to −17.70 kPa; 90° versus side, *p* = 0.002, MD = −101.56 kPa, and 95% CI = −164.43 to −38.70 kPa]. Furthermore, the PP were higher when performing 45° cutting then when conducting sideward cutting at the lesser toes region [*F*(2,14) = 4.955, *p* = 0.032; 45° versus side, *p* = 0.030, MD = 48.44 kPa, and 95% CI = 4.21–92.67 kPa] (Figure 2).

**Figure 3:**
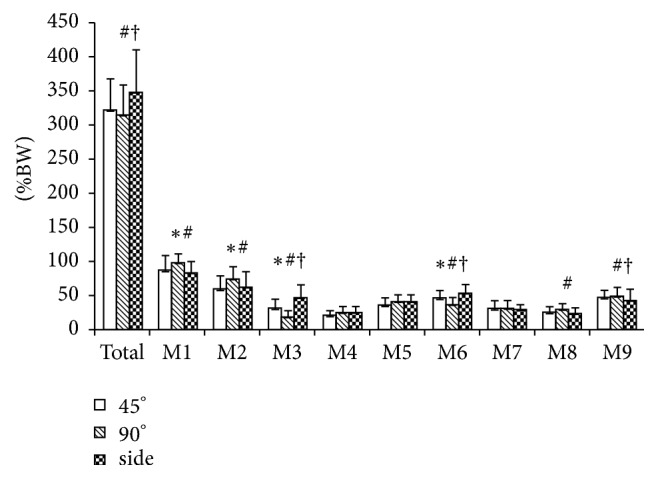
Comparison of maximum force among three cutting maneuvers. (A) M1, medial heel; M2, lateral heel; M3, medial midfoot; M4, lateral midfoot; M5, medial forefoot; M6, central forefoot; M7, lateral forefoot; M8, great toe; M9, lesser toes. (B) 90° cutting versus 45° cutting, ^*∗*^*p* < 0.05; 90° cutting versus sideward cutting, ^#^*p* < 0.05; 45° cutting versus sideward cutting, ^†^*p* < 0.05.

The MF value was higher when performing sideward cutting than when conducting 45° cutting and 90° cutting at the total foot [*F*(2,14) = 5.094, *p* = 0.022; side versus 45°, *p* = 0.021, MD = 26.28 %BW, and 95% CI = 3.60–48.95 %BW; side versus 90°, *p* = 0.024, MD = 33.08 %BW, and 95% CI = 3.98–62.18 %BW]. The MF value was higher when performing 90° cutting than when conducting 45° cutting and sideward cutting at the medial heel [*F*(2,14) = 9.709, *p* = 0.002; 90° versus 45°, *p* = 0.018, MD = 11.07 %BW, and 95% CI = 1.76–20.37 %BW; 90° versus side, *p* = 0.007, MD = 15.90 %BW, and 95% CI = 4.20–25.79 %BW]. The MF value was higher when performing 90° cutting than when conducting 45° cutting at the lateral heel [*F*(2,14) = 8.176, *p* = 0.004; 90° versus 45°, *p* = 0.002, MD = 14.56 %BW, and 95% CI = 5.19–23.93 %BW]. Moreover, the MF value was lower when performing 90° cutting than when conducting 45° cutting and sideward cutting at the medial midfoot [*F*(2,14) = 17.548, *p* < 0.001; 90° versus 45°, *p* < 0.001, MD = −12.22 %BW, and 95% CI = −18.76 to −5.68 %BW; 90° versus side, *p* < 0.001, MD = −28.64 %BW, and 95% CI = −41.26 to −16.03 %BW] and the central forefoot [*F*(2,14) = 35.385, *p* < 0.001; 90° versus 45°, *p* < 0.001, MD = −9.30 %BW, and 95% CI = −12.81 to −5.79 %BW; 90° versus side, *p* < 0.001, MD = −17.07 %BW, and 95% CI = −23.27 to −10.87 %BW]. Furthermore, the MF value was lower when performing 45° cutting than when conducting sideward cutting at the medial midfoot [*p* < 0.001, MD = −16.42 %BW, and 95% CI = −24.18 to −8.66 %BW] and the central forefoot [*p* = 0.008, MD = −7.77 %BW, and 95% CI = −13.55 to −1.99 %BW]. The MF value was lower when performing sideward cutting than when conducting 45° cutting and 90° cutting at the lesser toes [*F*(2,14) = 4.220, *p* = 0.037; side versus 45°, *p* = 0.044, MD = −6.33 %BW, and 95% CI = −12.53 to −0.14 %BW; side versus 90°, *p* = 0.038, MD = −7.42 %BW, and 95% CI = −14.49 to −0.35 %BW]. In addition, the MF value was higher when performing 90° cutting than when conducting sideward cutting at the great toes [*F*(2,14) = 7.743, *p* = 0.005; 90° versus side, *p* = 0.003, MD = 6.93 %BW, and 95% CI = 2.34–11.52 %BW] (Figure 3). Mean difference (95% CI) of plantar loads among recorded regions during three cutting tasks can be found in Table 2.

## 4. Discussion

The present study compared the in-shoe plantar loads during 45° cutting, 90° cutting, and sideward cutting. Our results provide important information for a substantial understanding of the plantar loading in typical basketball cutting maneuvers. Generally, sideward cutting and 45° cutting performed higher plantar pressures and maximal force compared with 90° cutting at the medial midfoot and the central forefoot. Plantar pressure and maximal force were higher when performing 90° cutting than when conducting 45° cutting and sideward cutting at the heel. The highest maximal force at the total foot was found during sideward cutting.

In previous studies, the researchers investigated characteristics of plantar loads during different maneuvers, including cutting, running, and jumping [15, 16, 18–21]. Wong et al. verified that maximal sideward cutting and 45° cutting performed higher peak pressure compared with 3.3 m/s running at the medial portion of the foot plantar. No significant difference was found on peak pressure between sideward cutting and 45° cutting [16]. Queen et al. validated that plantar loads were greater at the medial portion of the foot plantar when performing sideward cutting than when conducting crossover cutting [15, 21]. Their study was conducted among soccer players wearing football boots [15, 16, 21]. The plantar loading characteristic during cutting maneuvers is limited among basketball players.

Our data confirm that cutting maneuvers influence the plantar loads at specific functional regions at the plantar surface. The results are consistent with views in previous studies. The characteristics of plantar loads distribution depend on the specific movement [19, 20]. In the current study, 90° cutting showed high plantar loads at the heel and low plantar loads at the medial midfoot and the central forefoot. The characteristics may have resulted from the specific kinematics of cutting. The results indicated that the heel endured high plantar loads during 90° cutting. Consequently, the knee may endure greater vertical ground reaction force in the 90° cutting task than in other cutting tasks.

Several studies asserted that sideward cutting and 45° cutting performed higher plantar pressures in the medial portion compared with running [16, 22]. In the current study, sideward cutting and 45° cutting had higher peak pressure and maximal force at the medial midfoot and the central forefoot compared with 90° cutting. Furthermore, no significant differences on plantar pressure were found between sideward cutting and 45° cutting in the current study. The result is consistent with the findings of Wong et al. [16]. However, sideward cutting performed higher maximal force at the total foot, the medial midfoot, and the central forefoot compared with 45° cutting in current study. The result indicates that these areas may absorb extra ground reaction force. The difference in plantar force may be caused by different kinetic and kinematic adjustments for different cutting tasks. Furthermore, the differences in plantar forces may influence various sport injuries.

To our knowledge, many researches sought to reveal plantar pressure value in athletic individuals and its relevance to musculoskeletal injuries. Characteristics of plantar load distribution can provide guidance for emerging methods for musculoskeletal tissue repair and regeneration after sports injuries and help analyze athletic performance [23, 24]. The comparison of in-shoe plantar pressure values before and after sports injuries like ankle sprain and ACL injuries can be used to estimate the power generation and stability control of the foot. High and repetitive plantar loads may be one of the potential risk factors that cause chronic lower extremity injuries in athletes [13, 14]. Basketball players spend 31% of their playing time performing cutting maneuvers in different directions in an entire competitive basketball game [8]. Characteristics of plantar pressure distribution and magnitude during different maximum-effort cutting maneuvers suggest that effective methods should be developed to reduce high plantar loads and promote musculoskeletal tissue repair after injuries among basketball players. Appropriate training programs, injury prevention programs, rehabilitation programs, and footwear designs should be conducted to reduce plantar loads. The measurement of in-shoe plantar load can be used to evaluate the effects of specific training programs. The foot core stability related muscle training programs can be designed to improve foot functional performance [25]. It may reduce high plantar loads at the heel during 90° cutting as well as high plantar loads at the medial midfoot and the central forefoot during sideward cutting and 45° cutting. Using a soft midsole shoe condition in the forefoot region may be a plausible method to reduce the high plantar loads experienced by basketball players [19].

There are several limitations in this study. Other risk factors, such as the alignment of the lower limbs and footwear, may also influence the risk of sport injuries. These factors should be considered in future studies. Moreover, for an in-depth discussion of the consequences of cutting maneuvers on the plantar loads produced, a lower extremity kinematic analysis associated with the plantar loads should be conducted. These results may provide valuable information on lower extremity adjustment to different cutting maneuvers and, thus, contribute to more technologies for musculoskeletal tissue repair and regeneration after sport injuries in basketball players.

## 5. Conclusion

Plantar loads differed during the three maximum-effort cutting maneuvers. Sideward cutting and 45° cutting performed higher plantar pressures and maximal force compared with 90° cutting at the medial midfoot and the central forefoot. The highest plantar pressure and maximal force were found at the heel during 90° cutting. The highest maximal force at the total foot was found during sideward cutting. Differences in the plantar loads for different cutting tasks may be potential risks for overuse-related injuries to the lower extremities of basketball players.

## Figures and Tables

**Table 1:** Characteristics on plantar pressure during different maximum-effort cutting maneuvers.

	PP (kPa)				MF (%BW)			
	45° cutting	90° cutting	Sideward cutting		45° cutting	90° cutting	Sideward cutting	
Total	519.28 ± 89.13	521.47 ± 88.20	548.29 ± 83.16		321.75 ± 47.08	314.95 ± 43.93	348.02 ± 62.89	#†
M1	383.35 ± 67.39	427.70 ± 53.78	370.24 ± 89.97	*∗*#	88.14 ± 20.66	99.01 ± 13.74	83.31 ± 18.47	*∗*#
M2	360.11 ± 100.73	414.18 ± 86.25	346.09 ± 106.81	*∗*#	60.35 ± 19.62	74.92 ± 17.29	63.18 ± 23.28	*∗*#
M3	205.62 ± 88.43	138.64 ± 40.05	245.36 ± 103.74	*∗*#	31.05 ± 14.95	18.83 ± 10.53	47.47 ± 20.66	*∗*#†
M4	146.06 ± 36.41	144.50 ± 38.74	159.47 ± 53.11		20.37 ± 7.74	25.85 ± 10.35	25.68 ± 9.65	
M5	451.23 ± 132.87	472.47 ± 117.28	499.95 ± 120.74		35.67 ± 12.29	41.60 ± 10.53	41.69 ± 10.82	
M6	419.11 ± 120.67	357.45 ± 123.72	459.01 ± 113.47	*∗*#	47.28 ± 10.41	37.98 ± 10.44	55.05 ± 12.55	*∗*#†
M7	196.58 ± 45.91	194.12 ± 42.09	182.89 ± 39.99		32.92 ± 10.59	33.04 ± 9.63	30.87 ± 7.30	
M8	336.28 ± 103.68	376.92 ± 89.55	330.35 ± 87.14		26.22 ± 9.28	31.53 ± 8.78	24.60 ± 7.43	#
M9	330.30 ± 88.07	333.36 ± 81.08	281.87 ± 78.85	†	49.11 ± 9.99	50.20 ± 12.60	42.78 ± 12.05	#†

PP, peak pressures; MF, maximum force; M1, medial heel; M2, lateral heel; M3, medial midfoot; M4, lateral midfoot; M5, medial forefoot; M6, central forefoot; M7, lateral forefoot; M8, great toe; M9, lesser toes. 90° cutting versus 45° cutting, ^∗^*p* < 0.05; 90° cutting versus sideward cutting, ^#^*p* < 0.05; 45° cutting versus sideward cutting, ^†^*p* < 0.05.

**Table 2:** Mean difference (95% CI) of plantar loads among recorded regions during three cutting tasks.

	Region	45°–90°	90° side	45° side
PP (KPa)	Total	−2.19 (−22.12–17.74)	−26.82 (−75.97–22.34)	−29.01 (−71.17–13.15)
	M1	−44.35 (−86.53–−2.17)^*∗*^	57.47 (3.49–111.45)^#^	13.12 (−34.62–60.85)
	M2	−54.07 (−106.29–−1.86)^*∗*^	68.10 (1.86–134.34)^#^	14.02 (−39.82–67.86)
	M3	66.98 (8.65–125.32)^*∗*^	−106.72 (−178.99–−34.46)^#^	−39.74 (−100.35–20.87)
	M4	1.56 (−20.48–23.59)	−14.96 (−51.61–21.69)	−13.41 (−38.34–11.52)
	M5	−21.24 (−67.60–25.12)	−27.49 (−114.63–59.65)	−48.73 (−119.95–22.50)
	M6	61.66 (17.70–105.63)^*∗*^	−101.56 (−164.43–−38.70)^#^	−39.90 (−99.01–19.21)
	M7	2.46 (−18.13–23.05)	11.23 (−22.59–45.05)	13.69 (−7.60–34.97)
	M8	−40.64 (−99.27–17.99)	46.57 (−3.56–96.70)	5.93 (−36.99–48.85)
	M9	−3.06 (−49.56–43.45)	51.50 (−4.70–107.69)	48.44 (4.21–92.67)^†^

MF (%BW)	Total	6.81 (−12.37–25.98)	−33.08 (−62.18–−3.98)^#^	−26.28 (−48.95–−3.60)^†^
	M1	−11.07 (−20.37–−1.76)^*∗*^	15.90 (4.200–27.590)^#^	4.83 (−9.07–18.72)
	M2	−14.56 (−23.93–−5.19)^*∗*^	11.73 (−.89–24.36)	−2.83 (−13.16–7.50)
	M3	12.22 (5.68–18.76)^*∗*^	−28.64 (−41.26–−16.03)^#^	−16.42 (−24.18–−8.66)^†^
	M4	−2.78 (−7.79–2.22)	.174 (−5.80–6.15)	−2.61 (−6.89–1.68)
	M5	−5.93 (−11.16–.69)	−.094 (−8.70–8.51)	−6.019 (−12.36–.32)
	M6	9.30 (5.79–12.81)^*∗*^	−17.07 (−23.27–−10.87)^#^	−7.77 (−13.55–−1.99)^†^
	M7	−.12 (−4.60–4.36)	2.16 (−3.95–8.28)	2.04 (−2.64–6.73)
	M8	−5.31 (−10.98–.373)	6.93 (2.34–11.52)^#^	1.62 (−2.66–5.91)
	M9	−1.09 (−6.08–3.91)	7.421 (.35–14.49)^#^	6.334 (.14–12.53)^†^

PP, peak pressures; MF, maximum force; M1, medial heel; M2, lateral heel; M3, medial midfoot; M4, lateral midfoot; M5, medial forefoot; M6, central forefoot; M7, lateral forefoot; M8, great toe; M9, lesser toes. 90° cutting versus 45° cutting, ^*∗*^*p* < 0.05; 90° cutting versus sideward cutting, ^#^*p* < 0.05; 45° cutting versus sideward cutting, ^†^*p* < 0.05.
